# Translating the Burden of Pollen Allergy Into Numbers Using Electronically Generated Symptom Data From the Patient’s Hayfever Diary in Austria and Germany: 10-Year Observational Study

**DOI:** 10.2196/16767

**Published:** 2020-02-21

**Authors:** Katharina Bastl, Maximilian Bastl, Karl-Christian Bergmann, Markus Berger, Uwe Berger

**Affiliations:** 1 Aerobiology and Pollen Information Research Unit Department of Oto-Rhino-Laryngology Medical University of Vienna Vienna Austria; 2 Foundation German Pollen Information Berlin Germany; 3 Department of Dermatology, Venerology and Allerogology Charitè Universitätsmedizin Berlin Germany; 4 Paracelsus Medizinische Privatuniversität Salzburg Austria

**Keywords:** symptom data, Patient’s Hayfever Diary, pollen allergy, symptom score calculation

## Abstract

**Background:**

Pollen allergies affect a significant proportion of the population globally. At present, Web-based tools such as pollen diaries and mobile apps allow for easy and fast documentation of allergic symptoms via the internet.

**Objective:**

This study aimed to characterize the users of the Patient’s Hayfever Diary (PHD), a Web-based platform and mobile app, to apply different symptom score calculations for comparison, and to evaluate the contribution of organs and medications to the total score for the first time.

**Methods:**

The PHD users were filtered with regard to their location in Austria and Germany, significant positive correlation to the respective pollen type (birch/grass), and at least 15 entries in the respective season. Furthermore, 4 different symptom score calculation methods were applied to the datasets from 2009 until 2018, of which 2 were raw symptom scores and 2 were symptom load index (normalized) calculations. Pearson correlation coefficients were calculated pairwise for these 4 symptom score calculations.

**Results:**

Users were mostly male and belonged to the age groups of 21 to 40 years or >40 years. User numbers have increased in the last 5 years, especially when mobile apps were made available. The Pearson correlation coefficients showed a significant linear relationship above 0.9 among the 4 symptom score datasets and thus indicated no significant difference between the different methods of symptom score calculation. The nose contributed the most to the symptom score and determined about 40% of the score.

**Conclusions:**

The exact method of calculation of the symptom score is not critical. All computation methods show the same behavior (increase/decrease during the season). Therefore, the symptom load index is a useful computation method in all fields exploring pollen allergy, and Web-based diaries are a globally applicable tool to monitor the effect of pollen on human health via electronically generated symptom data.

## Introduction

### Background

Pollen allergy is an overreaction of the immune system to a foreign substance such as pollen grains or (free) allergens. This overreaction inflames the skin, sinuses, airways, or the digestive system [1]. The severity of allergies varies individually and may range from minor irritation to anaphylaxis. The most common symptoms of respiratory allergies are allergic rhinitis, allergic conjunctivitis, and asthma. Pollen allergy is a major problem globally [2] and affects a considerable percentage of the population ranging from 5% to 30% in industrialized countries [3]. The prevalence of pollen allergies is assumed to increase [4] along with its socioeconomic impact [2,5]. Furthermore, 1 million people of 8 million inhabitants in Austria are considered to be affected by pollen allergy [6], and almost 20% of the adults in Germany are affected by an allergy [7].

Only a minority of plants cause pollen allergies. Less than 100 species of 250,000 pollen-producing plants are of major interest in this respect [8-10]. For people with pollen allergy globally as well as in Austria and Germany, *Betula* (birch) and Poaceae (sweet grass family) are considered plants of high importance. Therefore, the birch and grass pollen seasons were selected in this study.

The allergenicity of pollen is influenced by climate, humidity, temperature, and air pollution [11]. The World Allergy Organization (WAO) recommends avoiding the main risk factors including outdoor air pollution [2,12]. Pollen itself may be seen as a *green pollutant*, and its occurrence in the air above a certain level or concentration may be regarded as an additional factor for air quality, comparable with the levels defined for sulfur dioxide, particulate matter, ozone, or nitrogen dioxide [12]. There is evidence that allergenicity, and thus the burden of allergy, increases with increased levels of air pollution [13-15]. However, allergen content and pollen concentrations are 2 different datasets and cannot always be compared with each other, especially because free allergens are not carried by pollen [16-19]. State-of-the-art pollen monitoring accounts for this fact and has to fulfill certain requirements to allow appropriate pollen information, for example, including symptom data to compensate for the lack of knowledge about the occurrence of major and minor allergens or personal exposure [20,21].

### Value of Electronically Generated Symptom Data

The idea of using symptom data in pollen information originates from clinical trials for immunotherapies for the treatment of allergic diseases including the feedback of those affected by pollen allergy for dose finding or confirmatory studies in the so-called symptom scores [22]. Most questionnaires of the freely available crowdsourced symptom diaries have a strong relation to the questionnaires of the European Medicines Agency (EMA) for such immunotherapy trials and should, therefore, be comparable but have not been evaluated for comparability so far.

However, scaling the burden is as important as allergen avoidance itself to improve and monitor the quality of life of the persons concerned: pollen forecasts and pollen information are valuable tools for support [23,24] and are strongly requested for during the pollen season [25]. Recently, pollen forecasts and pollen information have been distributed increasingly via mobile health (mHealth) technology such as mobile phones, tablets, and other wireless devices. The use of electronic health (eHealth) technology as a communication and information channel has gained significant importance to inform the public. This phenomenon is observed in countries with higher income [26]. The outreach via mHealth or eHealth technology allowed for symptom data to be used as a crowdsourced indication for the burden caused by pollen allergies and to monitor the impact of pollen on human health. Therefore, such data are integrated more often into pollen information besides pollen measurements and into studies dealing with pollen allergies. Working directly with patients is time consuming and not cost effective. Up to now, a number of internet tools and mobile apps are available based on country and technology [27-30].

### Crowdsourced User Data

The Patient’s Hayfever Diary (PHD), also called pollen diary, was first made available in 2009, developed by UB at the Medical University of Vienna. The pollen diary grew in terms of the included countries, available languages, and usability (available also as the mobile app, *Pollen*) as well as in user numbers since then. At present, the website is available in 13 countries (Austria, Germany, Switzerland, Great Britain, France, Spain, Slovenia, Sweden, Finland, Turkey, Hungary, Serbia, and Lithuania), whereas the mobile app is available in 8 countries/regions (Austria, Germany, Switzerland, Great Britain, France, Spain, Sweden, and South Tyrol in Italy). More than 240,000 users have entered data across Europe so far, with more than 32,000 users in Austria and more than 160,000 users in Germany over the whole period, making these 2 countries ideal for an in-depth study of electronically generated symptom data (data request on February 12, 2019). Symptom data retrieved from the pollen diary were already analyzed in a couple of studies [27,31]: Those show that an average based on a sufficiently high user number is robust and that symptom data give more insight into the onset of pollen allergy than pollen data alone.

### Objectives

The aims of this study were to (1) analyze the user profiles of the PHD, (2) perform an in-depth study for a 10-year dataset for 2 countries with the highest user numbers, and (3) apply and compare different symptom score calculations to judge their usability to monitor the effect of pollen on human health.

## Methods

### Patient’s Hayfever Diary

The PHD was used as a source for electronically generated symptom data. Data may derive from the webpage or mobile apps (*Pollen* or *Husteblume,* the latter only for Germany). The symptom data generated are crowdsourced and gained from users, not patients, because of privacy and data protection issues. Nonetheless, a couple of measures allow for high quality of generated data (see Symptom Data and Symptom Score Calculation Methods). Users were analyzed for the first time with regard to the frequency of certain age groups and gender (see Multimedia Appendices 1 and 2).

The following explanation of the technical background underlines the applicability of such a tool globally: The pollen diary runs a Java-based app on a server in a data center of the Medical University of Vienna. Data are stored in a Structured Query Language database, including a daily encrypted backup stored off-site. Users interact with the pollen diary via a multilingual Web user interface that can be used with any modern Web browser and currently supports 11 languages. In addition, the pollen diary provides a representational state transfer (REST)–based application programming interface (API), which is used by the *Pollen* app to provide nearly the same functionality as the Web user interface. The pollen diary gathers information via APIs from the European Aeroallergen Network (EAN) database (for displaying pollen loads compared with the user’s symptoms) and an internal data exchange platform, which provides forecasts for pollen and air quality parameters (used for creating personalized forecasts inside the pollen diary). Data gathered by the pollen diary are used (anonymized) in scientific studies and papers. Every communication is secured via HTTP secure/transport layer security (Web user interface and REST API), and access to the REST API is restricted by an internet protocol address, where possible.

Users are granted anonymity. The PHD fulfills the latest European Union (EU) regulation on data privacy (regulation EU 2016/679), adheres to the General Data Protection Regulation, Directive 95/46/EC, and Council of the EU of the EU for data protection, and collects only a minimum of personal data such as email address. Personal data such as birthday, medical conditions, address, or true name are not obligatory. Moreover, personal and symptom datasets are saved on separate servers to avoid any unauthorized connection between them.

### Symptom Data and Symptom Score Calculation Methods

The requirement for all users to be included in the study was based on their location (Austria and Germany). The PHD includes an automated background correlation service that correlates users to the pollen concentration of the respective region. For this study, only users with a significant positive correlation to the respective pollen type (birch or grass; *P*<.01 or *P*<.05) and 15 or more data entries within the respective pollen season (birch or grass) were included. This procedure limited the available symptom data but provided high-quality data of the symptom scores of users whose scores approach the scores of those diagnosed with pollen allergy the most.

A total of 4 different calculation methods of the symptom data have been applied to the dataset: (1) a raw symptom score (used automatically in the PHD), (2) the symptom load index (SLI) of that raw PHD score, (3) the EMA score, and (4) the SLI of the EMA raw score (Tables 1 and 2).

**Table 1:** Results of the calculation of the raw Patient’s Hayfever Diary symptom score and the raw European Medicines Agency symptom score per year, season, and country.

Country, allergen, and year			Patient’s Hayfever Diary symptom score	European Medicines Agency symptom score
**Austria**				
	***Betula***			
		2009	5.5	3.3
		2010	6.3	3.7
		2011	6.8	4.2
		2012	5.4	3.5
		2013	8.4	5.3
		2014	6.1	3.8
		2015	6.0	3.8
		2016	7.1	4.1
		2017	4.8	2.9
		2018	8.0	4.8
	**Poaceae**			
		2009	3.8	2.3
		2010	3.7	2.2
		2011	3.9	2.4
		2012	3.6	2.3
		2013	4.0	2.5
		2014	4.0	2.5
		2015	4.6	2.9
		2016	4.5	2.8
		2017	4.4	2.7
		2018	4.3	2.6
**Germany**				
	***Betula***			
		2009	9.4	5.6
		2010	6.5	4.2
		2011	7.7	4.6
		2012	5.0	3.6
		2013	8.1	4.9
		2014	6.5	3.8
		2015	6.7	4.0
		2016	6.5	3.7
		2017	4.8	2.9
		2018	8.2	4.8
	**Poaceae**			
		2009	4.6	2.5
		2010	4.4	2.9
		2011	4.0	2.5
		2012	4.5	2.9
		2013	4.6	3.0
		2014	5.1	3.1
		2015	5.2	3.3
		2016	4.6	4.3
		2017	4.8	2.9
		2018	4.7	2.9

The calculations of the first two methods are described in detail in the study by Bastl et al [27] but have been summarized in this study for a direct comparison. The PHD user process asks for 3 organs of interest: eyes, nose, and lungs. A severity score from 0 to 3 is possible for each organ, resulting in a maximum of 9 points for all organs with no discomfort (no problems)=0, low discomfort (mild problems)=1, moderate discomfort (moderate problems)=2, and strong discomfort (severe problems)=3. Furthermore, 4 specific symptoms per organ can be selected in addition to this general severity: itching, foreign body sensation, redness, and watering (for the eyes); itching, sneezing, running, and blocked (for the nose); and wheezing, shortness of breath, cough, and asthma (for the lungs). Asthma was included in the PHD; although we are aware that asthma is a disease or condition rather than a symptom, it commonly manifests together with allergic rhinitis [2] and therefore should be documented as well. All selected symptoms and the highest severity for each organ amounted so far to 21 points. Medication was included as well by a weighted medication score assigning more points for medications that affect more than one organ, for example, eye drops do have an effect on the eyes but not on the lungs, whereas tablets do influence all the organs. Eye medication gives a total of 1.8 points, with 1 point for eye drops or tablets, 0.5 for others, and 0.3 for homeopathic medicine. Nose medication gives a total of 2.05 points, with 1 point for nose drops or tablets, 0.25 for eye drops, 0.5 for others, and 0.3 for homeopathic medicine. Lung medication gives a total of 0.8 points, with 0.25 for tablets or others and 0.3 for homeopathic medicine. All medications together amount to 4.65, thus resulting in a total symptom score ranging from 0 to a maximum of 25.65. This score is the raw PHD symptom score that was automatically generated by the pollen diary. The PHD raw symptom score has been developed based on the (1) clinical standards of the General Hospital of Vienna (Austria) and (2) published knowledge at that time but has never been validated. However, it should be noted that a similar score has been validated as a reliable and valid instrument for observational studies and clinical trials and that symptom and medication scores are recommended as a primary outcome of clinical trials [32]. The scale and the inclusion of 3 organs are the same, but the specific symptoms (3 per organ vs 4 per organ for the raw PHD score) and the exact weighting of medication are different. The results of the raw PHD scores are listed in Table 1.

The SLI of this raw symptom score is calculated as an average of the same pool of users (filtered per location, correlation with certain aeroallergens, and number of entries within a certain time frame, as mentioned previously) and the raw PHD symptom score within a certain range from a minimum of 0 up to a maximum of 10. The SLI is thus a normalization of the PHD raw symptom score and was developed to compare crowdsourced symptom data of the PHD with other datasets in a clear and comprehensible way. It has been successfully applied, and its robustness has been proven in a couple of publications [27,31,33]. The results of the SLI scores based on the PHD raw symptom score are listed in Table 2.

The EMA raw symptom score is calculated based on the directive EMA/414476/2011 of the EMA.

Symptoms are rated on a 4-point scale that is comparable to the PHD raw symptom score, with absent symptoms=0, mild symptoms=1, moderate symptoms=2, and severe symptoms=3. The organs included are eyes and nose only (no lung symptoms). Two symptoms are included for eyes (tearing and itching/grittiness/redness), and 4 symptoms are included for nose (nasal itching, sneezing, rhinorrhea, and nasal obstruction). Therefore, the maximum EMA raw symptom score amounts to 12 points. The results of the EMA raw symptom scores are listed in Table 1.

The SLI of the EMA raw score is calculated based on the EMA raw symptom score data and thus considers only symptoms associated with eyes and nose. The results of the SLI based on the EMA raw symptom score are listed in Table 2. In addition, the percentage of the affected organ was calculated for the two SLI methods (Table 2).

**Table 2:** Results of the symptom load index calculations (traditional and European Medicines Agency symptom load index) per year, season, and country, including the percentages of the contribution of each affected organ and the medication score.

Country, allergen, and year				Traditional SLI^a^ calculation											European Medicines Agency SLI calculation				
				SLI		Eyes (%)		Nose (%)		Lungs (%)		Med^b^ (%)		SLI		Eyes (%)		Nose (%)	
**Austria**																			
	***Betula***																		
			2009		4.8		22		39		14		25		4.2		33		67
			2010		5.4		27		36		13		24		4.7		39		61
			2011		5.6		26		39		14		21		5.1		37		63
			2012		4.8		25		42		11		22		4.5		34		66
			2013		6.5		30		38		13		19		6.1		39		61
			2014		5.2		24		41		15		20		4.7		33		67
			2015		5.2		25		42		11		22		4.8		35		65
			2016		5.8		22		37		17		24		4.9		34		66
			2017		4.5		23		38		13		26		3.9		34		66
			2018		6.3		28		36		13		23		5.7		40		60
	**Poaceae**																		
			2009		3.7		25		37		8		30		3.1		36		64
			2010		3.6		22		41		10		27		3.1		32		68
			2011		3.8		21		44		11		22		3.2		31		69
			2012		3.6		23		46		12		24		3.2		31		69
			2013		3.9		23		42		10		25		3.4		32		68
			2014		3.9		23		43		9		25		3.4		32		68
			2015		4.3		25		41		10		24		3.8		35		65
			2016		4.1		24		41		13		22		3.7		34		66
			2017		4.1		23		39		11		27		3.5		34		66
			2018		4.1		23		40		12		25		3.5		33		67
**Germany**																			
	***Betula***																		
			2009		6.7		23		38		18		21		6.0		35		65
			2010		5.4		26		44		11		19		5.1		33		67
			2011		6.1		25		38		15		22		5.4		35		65
			2012		5.0		27		41		13		19		4.7		36		64
			2013		6.3		27		38		15		20		5.7		38		62
			2014		5.5		23		38		16		23		4.7		34		66
			2015		5.7		26		37		15		22		5.1		38		62
			2016		5.5		24		38		15		23		4.8		34		66
			2017		4.4		24		40		12		24		3.9		34		66
			2018		6.4		27		37		14		22		5.7		38		62
	**Poaceae**																		
			2009		4.6		28		30		12		30		3.5		46		54
			2010		4.2		22		47		11		20		3.8		27		73
			2011		3.8		25		41		10		24		3.4		35		65
			2012		4.1		24		44		11		21		3.8		32		68
			2013		4.3		26		42		12		21		3.9		34		66
			2014		4.5		23		40		13		24		4.0		33		67
			2015		4.6		25		41		12		22		4.2		34		66
			2016		4.3		25		42		11		22		3.9		33		67
			2017		4.4		23		41		13		23		3.8		33		67
			2018		4.4		23		42		12		23		3.8		32		68

^a^SLI: symptom load index.

^b^Med: medication score.

### Pollen Data

We followed the terminology recommended by Galán et al [34] for aerobiological data. Pollen data were selected only from pollen monitoring stations of known high quality, low occurrence of gaps, and wide geographical coverage during the study period of 10 years to allow a justified estimation for the whole of Austria and Germany. All stations included are listed in Multimedia Appendix 3, including their exact location and height above the sea level, with 17 stations for Austria and 28 for Germany. Pollen data were evaluated following the minimum recommendations of the European aerobiology community [35] and the EAN and were derived from automatic volumetric pollen and spore traps of the Hirst design [36]. The EAN standard pollen season definition was chosen, as percentage definitions are recommended for retrospective studies [37]. The season starts at 1% of the Annual Pollen Integral (APIn [34]) and ends at 95% of the APIn of the respective aeroallergen following this definition. The resulting birch and grass pollen seasons with their APIn are given in Multimedia Appendix 4.

### Statistics

The graphs and correlation computations were performed using the statistical software R 3.4.3 [38]. The graphs were drafted with the package ggplot2 [39]. The correlation computations were calculated for the comparison of 4 symptom score calculation methods (Tables 3 and 4). The Pearson correlation coefficients were computed pairwise for all symptom scores, the raw PHD symptom score, the SLI of the raw PHD symptom score, the EMA raw score, and the SLI of the EMA score. The Pearson correlation coefficient is a measure of linear correlation between 2 variables (with 1=total positive correlation; 0=no linear correlation, and −1=total negative linear correlation) and commonly used when a linear relationship is assumed. This method was chosen because it shows the strength of the relationship between the different score calculations. In addition, cause/effect are not relevant in this study as the goal was to examine possible differences between calculation methods. In the preanalysis, we recognized most coefficients achieving values of 0.99 when comparing the scores. Hence, we compared the difference between 2 days to remove a trend component because the symptom data are dependent on pollen data and, thus, follow a trend. The resulting coefficients were slightly lower but still strongly significant, with most values achieving 0.9 (Tables 3 and 4).

**Table 3:** Pearson correlation coefficients for the birch (*Betula*) pollen season for the 4 symptom score calculation methods for Austria and Germany from 2009 to 2018. Note the high correlation values for every comparison.

Country and year	EMA^a^ raw×EMA SLI^b^	EMA raw×PHD^c^	EMA raw×SLI	EMA SLI×PHD	EMA SLI×SLI	PHD×SLI
AT^d^ 2009	0.964	0.954	0.938	0.914	0.947	0.960
DE^e^ 2009	0.953	0.946	0.914	0.909	0.960	0.942
AT 2010	0.981	0.984	0.975	0.959	0.982	0.980
DE 2010	0.932	0.974	0.918	0.908	0.972	0.930
AT 2011	0.967	0.979	0.954	0.952	0.971	0.971
DE 2011	0.979	0.985	0.965	0.966	0.975	0.980
AT 2012	0.973	0.987	0.970	0.957	0.977	0.981
DE 2012	0.947	0.928	0.898	0.962	0.947	0.904
AT 2013	0.982	0.992	0.979	0.974	0.995	0.979
DE 2013	0.991	0.983	0.974	0.970	0.976	0.988
AT 2014	0.989	0.991	0.979	0.982	0.990	0.985
DE 2014	0.969	0.980	0.945	0.954	0.975	0.970
AT 2015	0.963	0.975	0.941	0.934	0.962	0.968
DE 2015	0.985	0.982	0.980	0.977	0.981	0.988
AT 2016	0.977	0.976	0.930	0.955	0.945	0.965
DE 2016	0.989	0.978	0.973	0.971	0.980	0.987
AT 2017	0.974	0.951	0.937	0.902	0.931	0.967
DE 2017	0.980	0.984	0.965	0.973	0.982	0.980
AT 2018	0.980	0.984	0.970	0.968	0.989	0.976
DE 2018	0.986	0.976	0.965	0.954	0.970	0.975

^a^EMA: European Medicines Agency.

^b^SLI: symptom load index.

^c^PHD: Patient’s Hayfever Diary.

^d^AT: Austria.

^e^DE: Germany.

**Table 4:** Pearson correlation coefficients for the grass (Poaceae) pollen season for the 4 symptom score calculation methods for Austria and Germany from 2009 to 2018. Note the high correlation values for every comparison.

Country and year	EMA^a^ raw×EMA SLI^b^	EMA raw×PHD^c^	EMA raw×SLI	EMA SLI×PHD	EMA SLI×SLI	PHD×SLI
AT^d^ 2009	0.954	0.956	0.913	0.914	0.934	0.953
DE^e^ 2009	0.878	0.823	0.789	0.725	0.830	0.903
AT 2010	0.963	0.977	0.946	0.937	0.970	0.957
DE 2010	0.948	0.950	0.929	0.886	0.955	0.943
AT 2011	0.971	0.974	0.950	0.937	0.960	0.969
DE 2011	0.953	0.963	0.929	0.908	0.958	0.940
AT 2012	0.959	0.972	0.926	0.927	0.960	0.950
DE 2012	0.956	0.971	0.913	0.938	0.954	0.951
AT 2013	0.983	0.983	0.969	0.961	0.975	0.978
DE 2013	0.973	0.975	0.948	0.943	0.962	0.966
AT 2014	0.969	0.972	0.940	0.937	0.958	0.965
DE 2014	0.965	0.965	0.951	0.923	0.959	0.965
AT 2015	0.974	0.971	0.958	0.940	0.968	0.972
DE 2015	0.963	0.971	0.940	0.926	0.956	0.962
AT 2016	0.966	0.957	0.935	0.922	0.959	0.959
DE 2016	0.985	0.991	0.975	0.977	0.990	0.983
AT 2017	0.965	0.962	0.923	0.924	0.949	0.950
DE 2017	0.971	0.980	0.950	0.950	0.974	0.968
AT 2018	0.968	0.963	0.939	0.913	0.939	0.957
DE 2018	0.973	0.974	0.944	0.945	0.954	0.970

^a^EMA: European Medicines Agency.

^b^SLI: symptom load index.

^c^PHD: Patient’s Hayfever Diary.

^d^AT: Austria.

^e^DE: Germany.

## Results

### User Characterization

In general, user numbers were low at the launch of the PHD and increased toward the last years (Multimedia Appendices 1 and 2). The average user numbers over the whole period of 10 years were higher in the grass pollen season than that in the birch pollen season. There was a notable increase in 2013, when the PHD became available as a mobile app (*Pollen*). The highest user numbers occurred in 2014 for the birch season and in 2015 for the grass pollen season in Austria. This is contrasted by the occurrence of the highest user numbers in 2016 for Germany for both the birch and the grass pollen seasons.

In the gender and age group distribution, less variation in different years could be observed. The gender distribution is fairly similar between Austria and Germany in both pollen seasons: Approximately 55% of users are male. It is noteworthy that the gender is usually indicated.

The age distribution (younger than 21 years, 21-40 years, older than 40 years, and unknown) was much less indicated by users, although only age groups was asked for and not a specific age or the birthday. Approximately 20% of users did not specify their age group on average. This applies to both countries and pollen seasons. The distribution to the aforementioned groups was fairly similar for Austria and Germany. Users younger than 21 years were the least frequent group, followed by the unknown age group. The most frequent age group varied for the birch and grass pollen seasons: The group older than 40 years dominated in the birch pollen season, whereas the group between the ages of 21 and 40 years dominated in the grass pollen season.

### Symptom Score Calculation Methods

**Figure 1:**
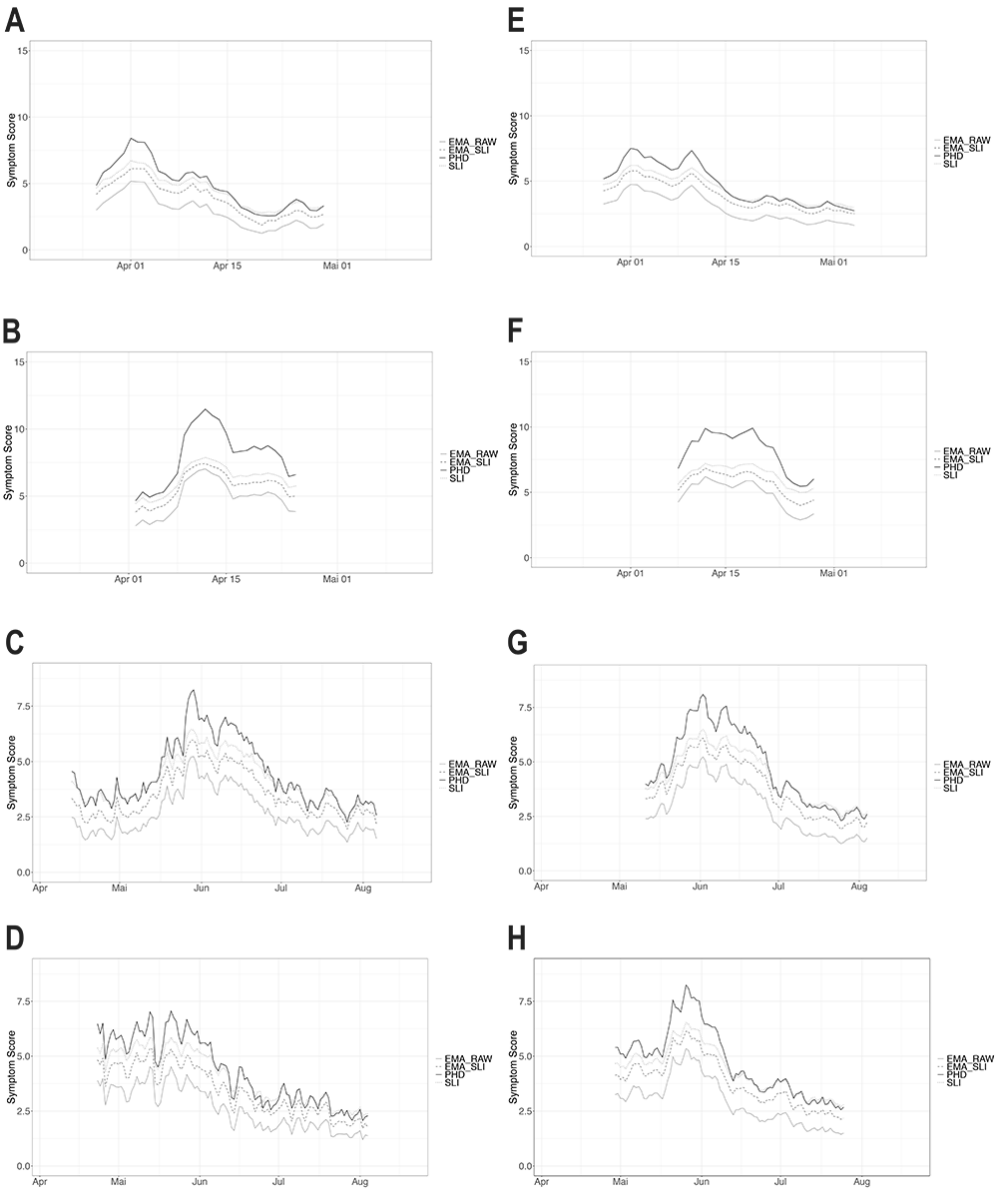
Pattern of the four calculation methods: dark continuous line=raw Patient’s Hayfever Diary score (PHD), gray dots=symptom load index of the raw Patient’s Hayfever Diary score (SLI), gray continuous line=European Medicines Agency raw score (EMA_RAW), gray dashed line=symptom load index of the EMA score (EMA_SLI) for the Austria (A-D) and Germany (E-H) for the birch (A-B and E-F) and grass (C-D and G-H) pollen seasons for the years 2017 (A, C, E, and G) and 2018 (B, D, F, and H).

The following patterns became apparent when comparing all score calculations in the period from 2009 to 2018 in Austria and Germany (Tables 1 and 2): (1) The scores were usually higher in the birch pollen season, (2) the scores varied from year to year (or season to season), and (3) the scores varied between the countries under study. The highest values were identified for the PHD raw score, followed by the SLI for the raw score, the SLI of the EMA score, and the raw EMA score. This was expected as the EMA raw scores included fewer symptoms and fewer organs, resulting in a lower maximum score. The raw scores resulted in low values in general. However, it has to be considered that these were computed averages and that experiencing the highest severity for all organs with all symptoms and medications is more than unrealistic for a relevant fraction of the population. The same pattern, for example, an increase or decrease of the score, can be observed between the 4 calculation methods. This behavior became even more apparent when visualized for 2017 and 2018 (Figure 1). The curves show the same course, and this applies to both countries, both pollen seasons, and all years. Only the relative level (absolute score values) varied because of the different calculation methods (Figure 1 and Multimedia Appendices 5-8).

The percentages calculated for the SLIs showed their relative contribution to the score (Table 2). These percentages represented a rather robust pattern for both the pollen seasons and the 2 countries. The variation can be attributed mostly to a yearly variation. The highest percentage value was attributed to the nose, followed by eyes, medication, and lungs. The importance of symptoms of the nose was emphasized when calculated for the SLI of the EMA score. The lung percentage was slightly higher during the birch pollen seasons, whereas the percentage for medication intake was slightly higher during the grass pollen seasons.

All computed Pearson correlations (Tables 3 and 4) were highly significant, showing the visually recognizable strong linear relationship between the series. The evident trend because of the relationship between symptom and pollen data series was removed from the time series.

## Discussion

### Overview

This study shows the evaluation of strictly filtered symptom data over 10 years in 2 Central European countries and pollen seasons. As such, it is informative for the symptom behavior and the user characterization in this region. In addition, 4 different symptom score calculation methods were applied to examine possible divergences in the results. The WAO recommends the inclusion of a concomitant symptom and medication score [40,41]. The PHD was developed based on this recommendation. Therefore, the PHD raw score and the resulting SLI included data on medication use. However, other score calculations were used as well, eg, those of the EMA that included only data on nose and eyes. Aerobiology and related fields often used nasal symptoms as a proxy, eg, nasal scores and medication use [42]; nose and eye symptoms with nose and eye medication [43]; nose and eye symptoms and a visual analog scale [44]; or eyes, nose, and lung symptoms without medication [24]. To our knowledge, the inclusion of nose symptoms applies to all symptom score calculations for pollen allergies.

### Principal Findings and Relation to Previous Work

It is worth discussing that our results challenge the current dogma of using a combined symptom and medication score. It seems that scoring symptoms gives the most information, but any indication from medication is missing. This might still be important for clinical trials. An analysis of symptoms vs symptoms and medication scores for clinical trials showed that both measures are able to verify the difference between the placebo and the group receiving the active substance [45]. However, the symptom score leads to less severe values than the score considering rescue medication [45]. The conclusion of that study was that a combined score is a valuable alternative and that the inclusion of rescue medication use leads to an improvement in assessing the symptom severity and treatment effect. Our study focused only on the relationship between the scores without any relation to treatment. Therefore, we cannot give recommendations concerning clinical trials, but for observational studies and the aerobiological field, the use of a symptom or a combined symptom and medication score is justified, as suggested by our data.

The calculation of the percentage regarding the contribution for specific organs and the medication intake showed a value of about 40% on average for the nose in this study. This pattern is visible for 10 years in 2 different pollen seasons and for 2 countries. Thus, the nose is recognized as the most important organ reporting allergic symptoms representing the main burden of a pollen allergy. These findings underline and complement previous studies concerning the significance of nose symptoms [46]. The organ *eyes* represents the second highest contribution to the main burden, directly followed by medication use. The additional use of one or the other is justified when analyzing symptom scores because of the similar contribution of both datasets. The lung symptoms contribute the least to the total score. This outcome is probably attributed to the fact that lung symptoms are not frequently experienced in most people affected by a pollen allergy [46].

### Lessons Learned and Limitations

The 4 different symptom score calculation methods underpin the value of nose symptoms for any symptom score. The progress and pattern (increase/decrease during the season) are corresponding in all calculations, although on a different level depending on the maximum scale for the respective score studied herein (Figure 1 and Multimedia Appendices 5-8). The Pearson correlation coefficients show a significant linear relationship between all symptom score calculation methods (Tables 3 and 4). Most values reach 0.9 even when calculated as the difference between days excluding the trend component (the dependence of symptom data on pollen concentrations). Most values below 0.9 occurred in the first year of the launch of the PHD (in 2009) when user numbers were low and not significant for such analyses.

Data on the user characterization of the PHD are presented herein for the first time and give valuable insights: user numbers are higher during the grass pollen season (Multimedia Appendix 2). Grass pollen allergy is the most frequent pollen allergy in east Austria [47], Germany [48], and Europe in general [4]. User numbers showed a significant increase when mobile apps were provided, which included the PHD as an additional service. This is evidenced by the launch of the mobile app, *Husteblume*, in 2016 in Germany and the launch of the *Pollen* app in 2013 in Austria and the introduction of personalized pollen information in 2014. The increase in user numbers was observed for both the birch and the grass pollen season. Moreover, nearly all users indicated their gender, but a relevant fraction of them did not indicate their age group. We observed that the PHD users are mostly male (60%:40% on average), and thus, the results are biased toward male (and German speaking because of the country selection) users. This finding should be taken into consideration for all conclusions and comparisons with the general population. The bias toward males could be explained by the behavior regarding the use of mobile technologies and the internet in general. Recent studies indicate that internet consumption by men is higher than that by women, even when accounting for age and ethnicity, with younger people using the internet most [49]. Moreover, internet use is higher in younger people and much lower in those aged older than 45 years, even more so in older adults (aged >65 years) who are less likely to adopt the internet [50]. The observation of sex differences (not performed in this study) could lead to a gender bias, especially in an unbalanced sample [51]. Therefore, we have restricted our findings to our user pool in total (females and males) and have to leave possible differences and inferences open to future studies. Our findings underline the importance of mHealth technology as a mobile communication channel [52].

The most indicated age group for the birch pollen season is those older than 40 years, contrasting with the results of the grass pollen season where most frequent users were in the age group of 21 to 40 years. This pattern was recognized in both countries analyzed in this study. It remains unknown why the user age groups differ between the two pollen seasons and which age group might be *hidden* most in the age group *unknown* and for what reasons.

Finally, the data give more evidence on spatiotemporal aspects of symptom data. Observations of higher and lower symptom score calculations for different years and pollen seasons (Tables 1 and 2) provide more evidence that the burden of those affected by pollen allergy varies [27]. There are less or more intense seasons and years in terms of the severity of symptoms of those possibly affected by pollen allergy. The biogeographical component is obscured because the analyses were performed on a country level. Still, it is evident that there are also geographical differences and small variations between the datasets from Austria and Germany. The grass pollen season seems to have an additional burden on average in Germany (Table 1), whereas the pattern of increase or decrease of the birch pollen seasons deviates between the 2 countries (eg, in 2015 and 2016; Table 1).

### Conclusions

Users of the PHD and its mobile apps are mostly male belonging to the age groups of 21 to 40 years (grass pollen season) or >40 years (birch pollen season). Crowdsourced symptom datasets can be seen as beneficial in terms of increasing the number of users of mHealth and eHealth technology and the availability of mobile apps: Users receive personalized information based on their individual symptoms and researchers gain insight into the real burden of those affected by pollen allergy. The user pool for Austria and Germany is fairly similar. The technique of a Web-based diary can be applied globally to allow international monitoring of the effect of pollen on human health.

The evaluation of 4 different symptom score calculations for 2 countries (Austria and Germany) and 2 pollen seasons (birch and grass) over the last decade showed that the choice of the calculation method is not critical. The inclusion of the nose as an affected organ and its symptoms is most relevant, as its contribution to the score calculation is the highest. Herein, the medication score is of similar importance as the eye symptom data. However, the Pearson correlation coefficients show a significant linear relationship for all calculation methods. The SLI calculations smoothen the pattern (and curves; see Figure 1) and give a more stable pattern when compared with the raw score calculations with fewer high or low values. Therefore, the SLI can be recommended as a symptom score calculation method for all apps such as clinical trials, but it points to the fact that all of the computation methods tested herein work as long as they are clearly defined, are consequently used, and include nose symptoms.

There is variation in the symptom scores between pollen seasons, years, and countries. Thus, studies should also refer to a comparison dataset to explore if their findings can be explained because of a known higher burden (specific pollen season), a strong season (year), sample-specific reaction pattern (gender, age group, and other parameters), or because of biogeographical factors (country/region).

Symptom data are a most valuable data source for aerobiology, allergology, and all fields involved in pollen allergy research because they give a direct indication about the burden of persons affected. Nonetheless, standardization of symptom scores is needed for clinical trials and allergology in general and should be the goal of a joint effort from all institutions and organizations concerned.

## Appendix

Multimedia Appendix 1 Characterization of user data from the Patient’s Hayfever Diary during the calculated birch (Betula) pollen season in Austria and Germany. Total user numbers, the percentage of gender (male/female/unknown), and the percentage of age groups (below 21 years /21-40 years/above 40 years/unknown) are presented per year and as an average of the 10 years of study period.

Multimedia Appendix 2 Characterization of user data from the Patient’s Hayfever Diary during the calculated grass (Poaceae) pollen season in Austria and Germany. Total user numbers, the percentage of gender (male/female/unknown), and the percentage of age groups (below 21 years/21-40 years/above 40 years/unknown) are presented per year and as an average of the 10 years of study period.

Multimedia Appendix 3 List of pollen monitoring stations included in this study for Austria and Germany and their exact location data and height above sea level. Pollen data were used only to calculate the respective pollen season and the Annual Pollen Integral.

Multimedia Appendix 4 Calculation of the Annual Pollen Integral and the pollen season for birch (Betula) and grasses (Poaceae) for Austria and Germany during 2009 until 2018.

Multimedia Appendix 5 Pattern of the four calculation methods: dark continuous line=raw Patient’s Hayfever Diary score (PHD), gray dots=symptom load index of the raw Patient’s Hayfever Diary score (SLI), gray continuous line=European Medicines Agency raw score (EMA_RAW), gray dashed line=symptom load index of the European Medicines Agency score (EMA_SLI) for Austria (A-D) and Germany (E-H) for the birch (A-B and E-F) and grass (C-D and G-H) pollen seasons for the years 2015 (A, C, E, and G) and 2016 (B, D, F, and H).

Multimedia Appendix 6 Pattern of the four calculation methods: dark continuous line=raw Patient’s Hayfever Diary score (PHD), gray dots=symptom load index of the raw Patient’s Hayfever Diary score (SLI), gray continuous line=European Medicines Agency raw score (EMA_RAW), gray dashed line=symptom load index of the European Medicines Agency score (EMA_SLI) for Austria (A-D) and Germany (E-H) for the birch (A-B and E-F) and grass (C-D and G-H) pollen seasons for the years 2013 (A, C, E, and G) and 2014 (B, D, F, and H).

Multimedia Appendix 7 Pattern of the four calculation methods: dark continuous line=raw Patient’s Hayfever Diary score (PHD), gray dots=symptom load index of the raw Patient’s Hayfever Diary score (SLI), gray continuous line=European Medicines Agency raw score (EMA_RAW), gray dashed line=symptom load index of the European Medicines Agency score (EMA_SLI) for Austria (A-D) and Germany (E-H) for the birch (A-B and E-F) and grass (C-D and G-H) pollen seasons for the years 2011 (A, C, E, and G) and 2012 (B, D, F, and H).

Multimedia Appendix 8 Pattern of the four calculation methods: dark continuous line=raw Patient’s Hayfever Diary score (PHD), gray dots=symptom load index of the raw Patient’s Hayfever Diary score (SLI), gray continuous line=European Medicines Agency raw score (EMA_RAW), gray dashed line=symptom load index of the European Medicines Agency score (EMA_SLI) for Austria (A-D) and Germany (E-H) for the birch (A-B and E-F) and grass (C-D and G-H) pollen seasons for the years 2009 (A, C, E, and G) and 2010 (B, D, F, and H).

## Abbreviations

API: application programming interface
APIn: Annual Pollen Integral
EAN: European Aeroallergen Network
eHealth: electronic health
EMA: European Medicines Agency
EU: European Union
mHealth: mobile health
PHD: Patient’s Hayfever Diary
REST: representational state transfer
SLI: symptom load index
WAO: World Allergy Organization
