# Supplementary material for: Translating the Burden of Pollen Allergy Into Numbers Using Electronically Generated Symptom Data From the Patient’s Hayfever Diary in Austria and Germany: 10-Year Observational Study
Source: J Med Internet Res. 2020 Feb 21;22(2):e16767. doi: 10.2196/16767 (PMC7060495; doi:10.2196/16767)
Supplement: Multimedia Appendix 1 [file jmir_v22i2e16767_app1.pdf]

| Characterization *Betula* | Austria | Germany |
|---|---|---|
| 2009 | | |
| Users total | 85 | 5 |
| Percentage gender | 53/46/1 | 40/60/0 |
| Percentage age groups | 2/39/49/9 | 0/40/40/20 |
| 2010 | | |
| Users total | 82 | 20 |
| Percentage gender | 61/39/0 | 75/25/0 |
| Percentage age groups | 5/27/54/15 | 15/40/35/10 |
| 2011 | | |
| Users total | 158 | 80 |
| Percentage gender | 58/42/0 | 52/48/0 |
| Percentage age groups | 8/28/43/21 | 5/29/48/19 |
| 2012 | | |
| Users total | 132 | 54 |
| Percentage gender | 64/36/0 | 56/44/0 |
| Percentage age groups | 5/35/34/27 | 9/24/54/13 |
| 2013 | | |
| Users total | 195 | 180 |
| Percentage gender | 67/33/0 | 56/44/0 |
| Percentage age groups | 5/36/38/21 | 5/28/40/27 |
| 2014 | | |
| Users total | 239 | 255 |
| Percentage gender | 57/43/0 | 67/33/0 |
| Percentage age groups | 5/32/36/28 | 7/31/36/26 |
| 2015 | | |
| Users total | 111 | 140 |
| Percentage gender | 58/42/0 | 65/35/0 |
| Percentage age groups | 4/31/42/23 | 4/29/24/43 |
| 2016 | | |
| Users total | 238 | 509 |
| Percentage gender | 62/38/0 | 51/49/0 |
| Percentage age groups | 6/32/39/23 | 6/37/43/14 |
| 2017 | | |
| Users total | 121 | 412 |
| Percentage gender | 59/41/0 | 57/43/0 |
| Percentage age groups | 10/27/44/19 | 7/42/45/6 |
| 2018 | | |
| Users total | 104 | 168 |
| Percentage gender | 56/44/0 | 53/47/0 |
| Percentage age groups | 8/29/58/6 | 10/39/49/2 |
| **Average 2009-2018** | | |
| **Users total** | **147** | **182** |
| **Percentage gender** | **60/40/0** | **57/43/0** |
| **Percentage age groups** | **6/32/44/18** | **7/34/41/18** |
