# Supplementary material for: Translating the Burden of Pollen Allergy Into Numbers Using Electronically Generated Symptom Data From the Patient’s Hayfever Diary in Austria and Germany: 10-Year Observational Study
Source: J Med Internet Res. 2020 Feb 21;22(2):e16767. doi: 10.2196/16767 (PMC7060495; doi:10.2196/16767)
Supplement: Multimedia Appendix 2 [file jmir_v22i2e16767_app2.pdf]

Characterization (Poaceae)	Austria	Germany
2009		
Users total	123	16
Percentage gender	58/40/2	56/44/0
Percentage age groups	6/39/44/11	6/50/38/6
2010		
Users total	169	53
Percentage gender	60/40/0	57/43/0
Percentage age groups	10/30/43/17	13/55/26/6
2011		
Users total	229	126
Percentage gender	60/40/0	60/40/0
Percentage age groups	12/34/33/20	11/35/31/23
2012		
Users total	180	160
Percentage gender	63/37/0	55/45/0
Percentage age groups	3/47/26/24	10/35/34/21
2013		
Users total	272	248
Percentage gender	60/40/0	57/43/0
Percentage age groups	7/43/29/21	14/37/27/22
2014		
Users total	246	256
Percentage gender	63/37/0	65/35/0
Percentage age groups	7/37/22/33	7/37/28/28
2015		
Users total	252	571
Percentage gender	58/42/0	55/45/0
Percentage age groups	9/38/25/28	5/18/15/61
2016		
Users total	163	1329
Percentage gender	57/43/0	52/48/0
Percentage age groups	7/41/26/26	9/52/33/5
2017		
Users total	188	754
Percentage gender	66/34/0	56/44/0
Percentage age groups	19/35/31/16	12/46/38/5
2018		
Users total	176	702
Percentage gender	59/41/0	56/44/0
Percentage age groups	15/37/44/4	9/45/45/1
**Average 2009-2015**		
**Users total**	**200**	**422**
**Percentage gender**	**60/39/1**	**57/43/0**
**Percentage age groups**	**10/38/32/20**	**10/41/32/17**
