# Supplementary material for: Translating the Burden of Pollen Allergy Into Numbers Using Electronically Generated Symptom Data From the Patient’s Hayfever Diary in Austria and Germany: 10-Year Observational Study
Source: J Med Internet Res. 2020 Feb 21;22(2):e16767. doi: 10.2196/16767 (PMC7060495; doi:10.2196/16767)
Supplement: Multimedia Appendix 3 [file jmir_v22i2e16767_app3.pdf]

Pollen-monitoring station	Latitude	Longitude	Height above sea level (m)
Allensteig (AT)	48.69139	15.36722	596
Freistadt (AT)	48.51361	14.50667	560
Graz (AT)	47.06417	15.43333	365
Innsbruck (AT)	47.28000	11.37861	620
Klagenfurt (AT)	46.63056	14.30556	446
Krimml (AT)	47.22211	12.17689	1067
Linz (AT)	48.30000	14.28333	266
Obergurgl (AT)	46.87861	11.01750	1930
Oberpullendorf (AT)	47.50333	16.50444	251
Reutte (AT)	47.46667	10.71667	853
St. Veit im Pongau (AT)	47.32629	13.14583	743
Tamsweg (AT)	47.13333	13.78333	1021
Vöcklabruck (AT)	48.00222	13.65806	452
Wien (AT)	48.24889	16.35611	209
Woergl (AT)	47.51111	12.07861	510
Zams (AT)	47.15444	10.59333	772
Zell am See (AT)	47.33056	12.81278	764
Aukrug (DE)	54.08528	09.82028	40
Berlin (DE)	52.52750	13.37806	39
Bochum (DE)	51.46667	07.18333	45
Bonn (DE)	50.73333	07.10000	105
Chemnitz (DE)	50.83333	12.91667	418
Delmenhorst (DE)	53.05083	08.63278	10
Dresden (DE)	51.05000	13.75000	230
Flensburg (DE)	54.78333	09.45000	33
Freiburg (DE)	48.00000	07.86667	270
Fulda (DE)	50.55000	09.68333	450
Goettingen (DE)	51.53333	09.95000	167
Greifswald (DE)	54.10000	13.40000	2
Bad Lippspringe (DE)	51.78333	08.81667	160
Loewenstein (DE)	49.10000	09.40000	350
Marburg (DE)	50.81472	08.80583	380
Moenchen Gladbach (DE)	51.20000	06.43333	38
Muenchen (DE)	48.13333	11.58333	535
Muennerstadt (DE)	50.25000	10.20000	260
Neustrelitz (DE)	53.36667	13.08333	70
Neustadt/Südharz (DE)	51.55861	10.83639	47
Prerow/Darß (DE)	54.45000	12.58333	8
Rostock (DE)	54.10000	12.15000	4
Soest (DE)	51.57528	08.11194	96
Sylt (Westerland) (DE)	54.92583	08.31500	12
Treuenbrietzen (DE)	52.10000	12.86667	70
Wangen (DE)	47.70000	09.83333	710
Westerland/Sylt (DE)	53.07278	08.51306	26
Westerloge (DE)	53.07500	08.51528	28
