# Supplementary material for: Translating the Burden of Pollen Allergy Into Numbers Using Electronically Generated Symptom Data From the Patient’s Hayfever Diary in Austria and Germany: 10-Year Observational Study
Source: J Med Internet Res. 2020 Feb 21;22(2):e16767. doi: 10.2196/16767 (PMC7060495; doi:10.2196/16767)
Supplement: Multimedia Appendix 4 [file jmir_v22i2e16767_app4.pdf]

**Region**	**Aeroallergen**	**Year**	**Season Start**	**Season End**	**APIn**
Austria					
	Betula	2009	05.04.2009	11.05.2009	3204
		2010	03.04.2010	02.05.2010	5831
		2011	29.03.2011	24.04.2011	3959
		2012	25.03.2012	29.04.2012	9061
		2013	15.04.2013	05.05.2013	4944
		2014	21.03.2014	23.04.2014	5669
		2015	07.04.2015	04.05.2015	3225
		2016	30.03.2016	03.05.2016	6401
		2017	27.03.2017	29.04.2017	4023
		2018	02.04.2018	25.04.2018	8120
	Poaceae	2009	25.04.2009	10.08.2009	2122
		2010	04.05.2010	04.08.2010	2678
		2011	22.04.2011	11.08.2011	2881
		2012	30.04.2012	17.08.2012	2100
		2013	02.05.2013	10.08.2013	2911
		2014	06.04.2014	11.08.2014	1850
		2015	29.04.2015	03.08.2015	3450
		2016	14.04.2016	14.08.2016	2933
		2017	13.04.2017	06.08.2017	2896
		2018	23.04.2018	04.08.2018	3082
Germany					
	Betula	2009	04.04.2009	24.04.2009	6485
		2010	02.04.2010	30.04.2010	7975
		2011	30.03.2011	28.04.2011	5500
		2012	25.03.2012	02.05.2012	4481
		2013	17.04.2013	06.05.2013	6475
		2014	22.03.2014	24.04.2014	11388
		2015	10.04.2015	03.05.2015	2918
		2016	05.04.2016	07.05.2016	8120
		2017	28.03.2017	04.05.2017	4750
		2018	08.04.2018	28.04.2018	8733
	Poaceae	2009	29.04.2009	19.08.2009	1828
		2010	14.05.2010	05.08.2010	1638
		2011	26.04.2011	06.08.2011	1637
		2012	10.05.2012	14.08.2012	1815
		2013	16.05.2013	04.08.2013	1972
		2014	01.05.2014	27.07.2014	2435
		2015	10.05.2015	08.08.2015	2440
		2016	09.05.2016	26.07.2016	2639
		2017	11.05.2017	04.08.2017	1948
		2018	29.04.2018	25.07.2018	2717
