# Supplementary figures and images for: Translating the Burden of Pollen Allergy Into Numbers Using Electronically Generated Symptom Data From the Patient’s Hayfever Diary in Austria and Germany: 10-Year Observational Study
Source: J Med Internet Res. 2020 Feb 21;22(2):e16767. doi: 10.2196/16767 (PMC7060495; doi:10.2196/16767)

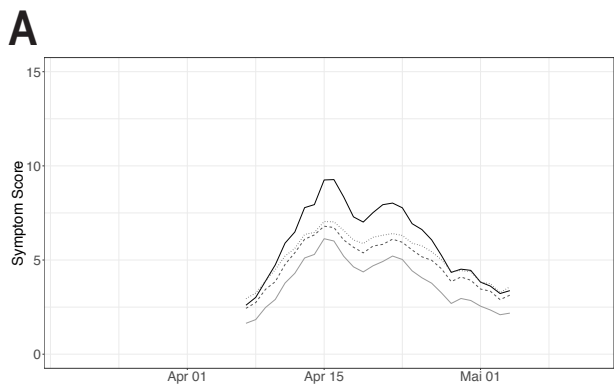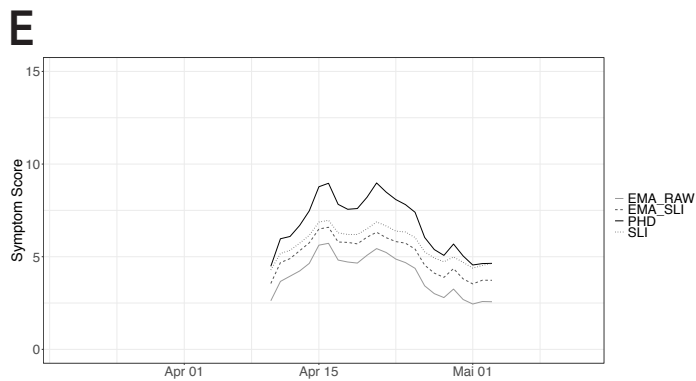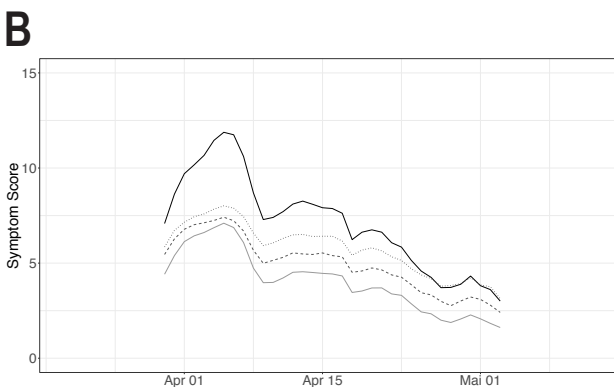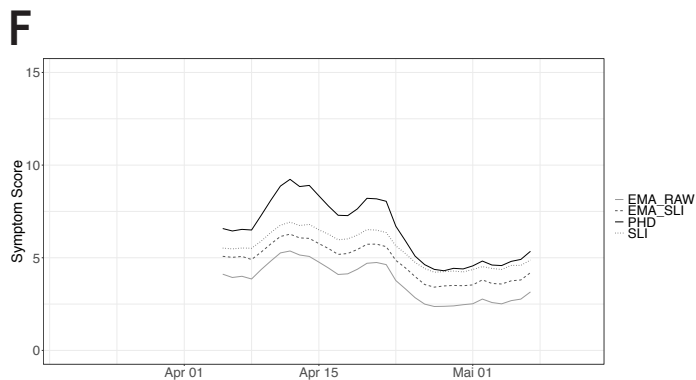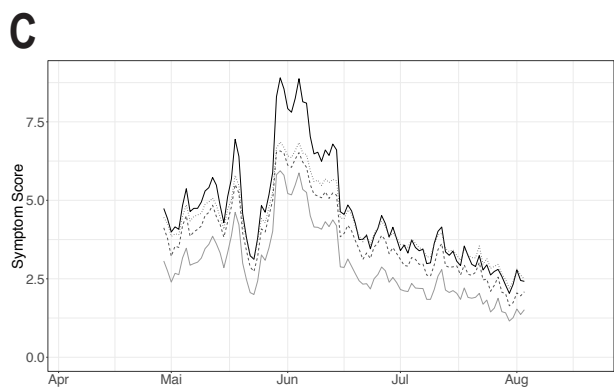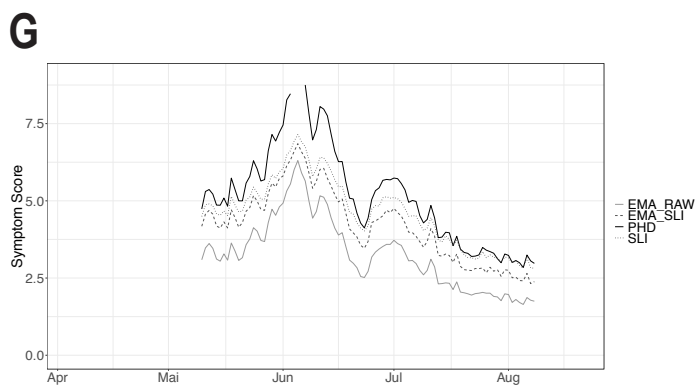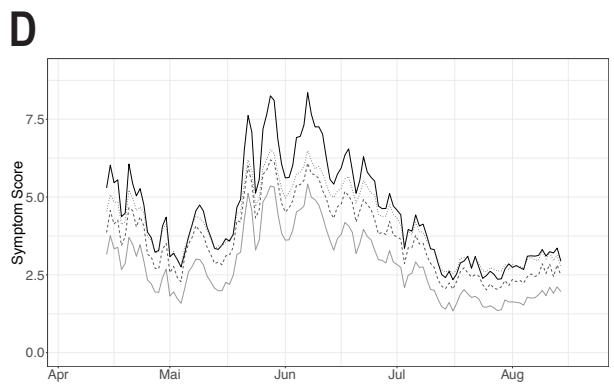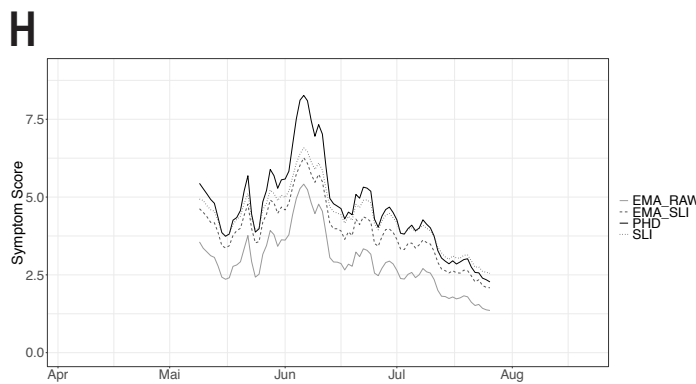

Supplement: Multimedia Appendix 5 [file jmir_v22i2e16767_app5.pdf]

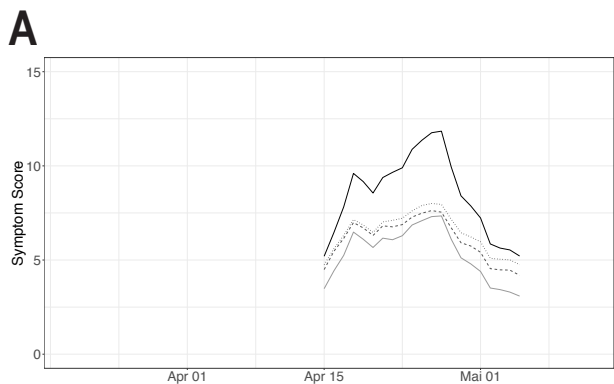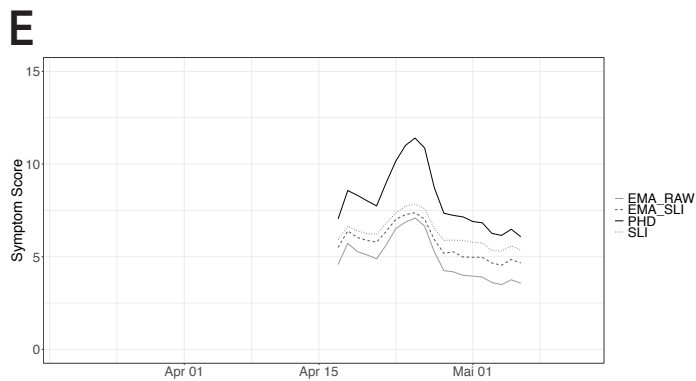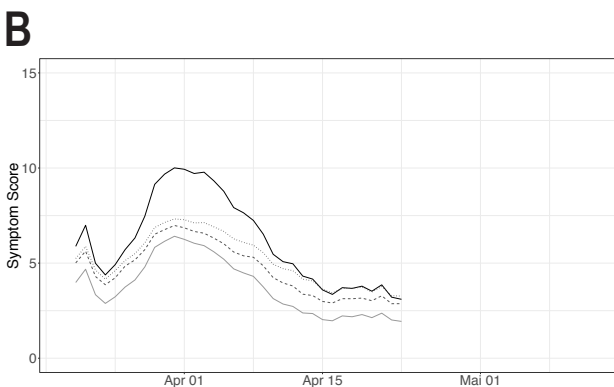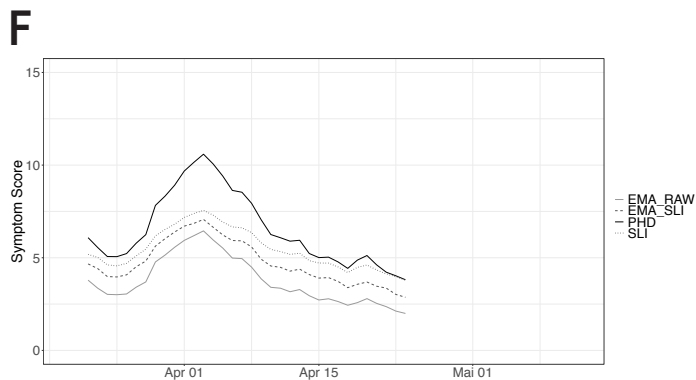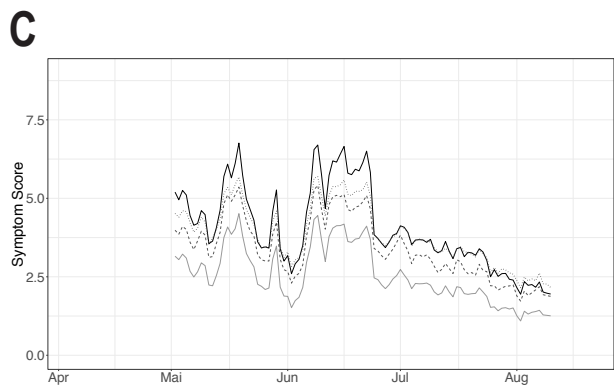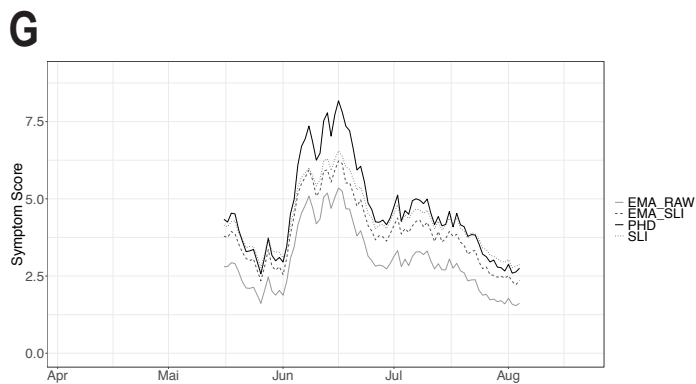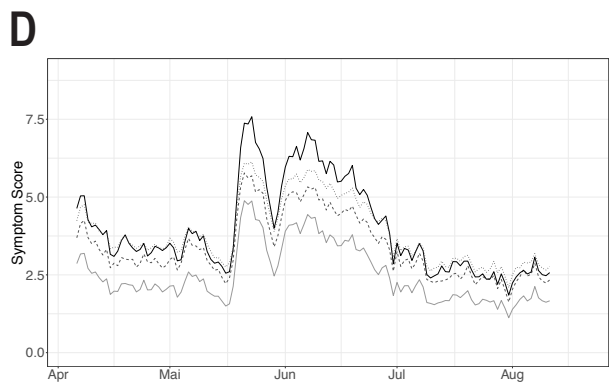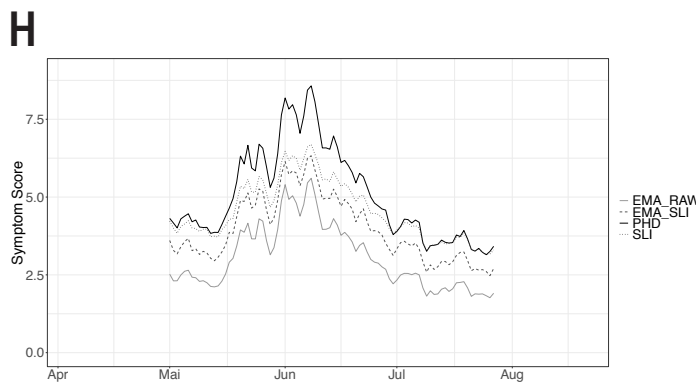

Supplement: Multimedia Appendix 6 [file jmir_v22i2e16767_app6.pdf]

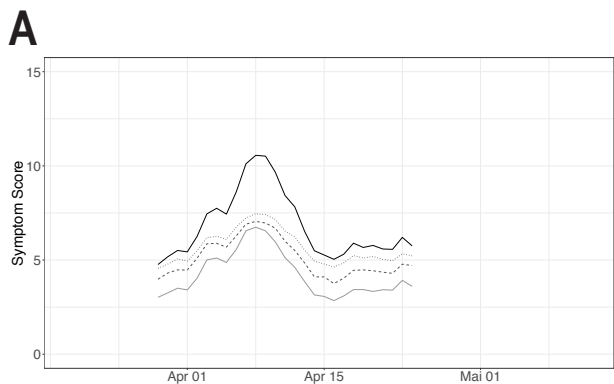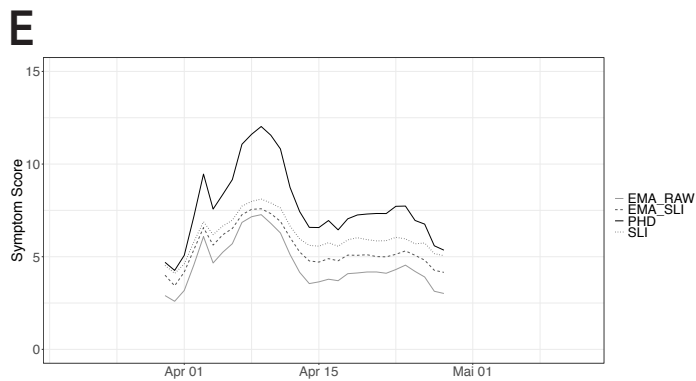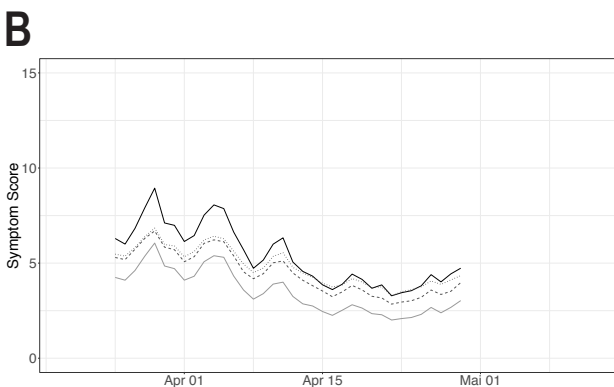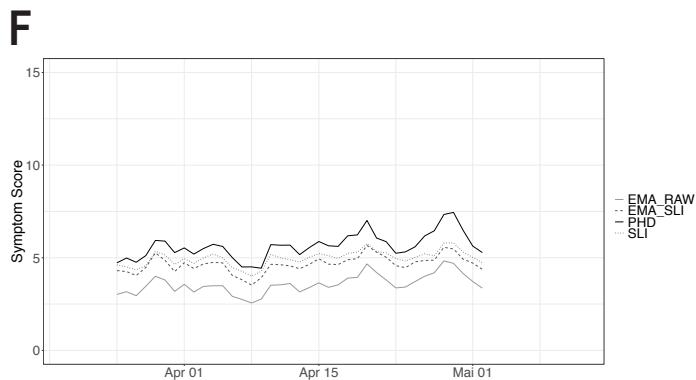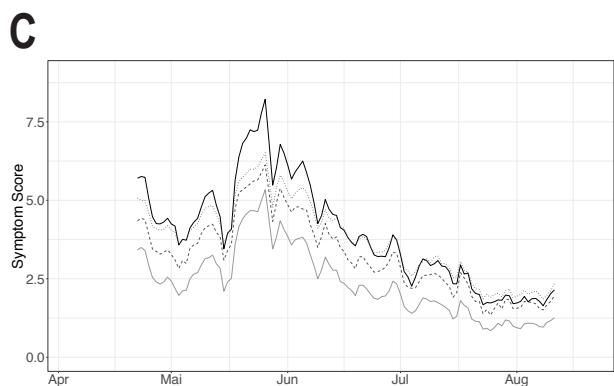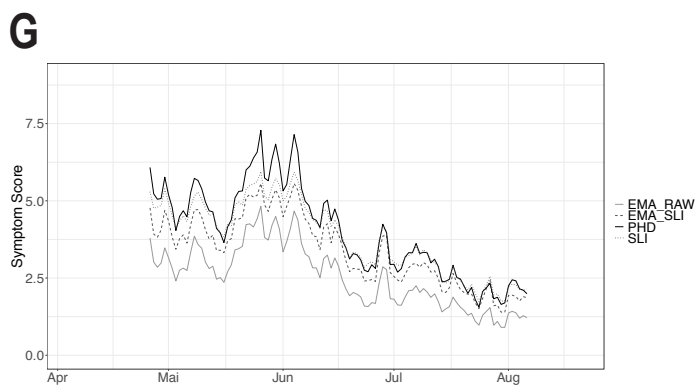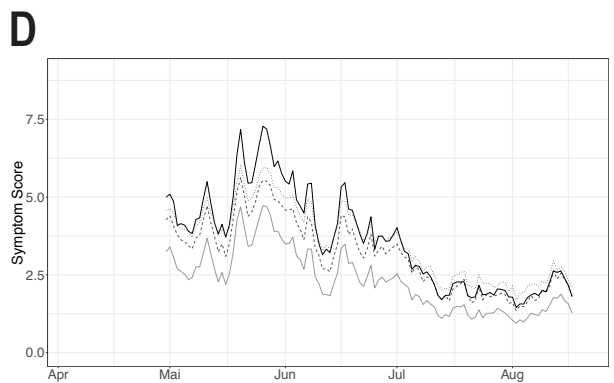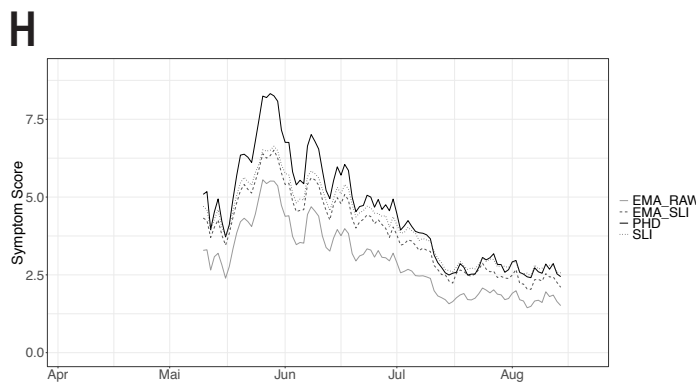

Supplement: Multimedia Appendix 7 [file jmir_v22i2e16767_app7.pdf]

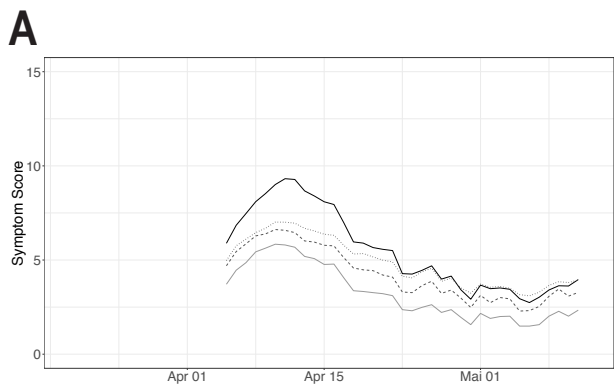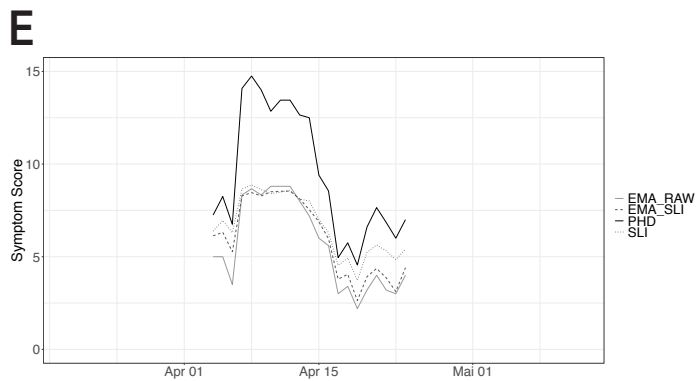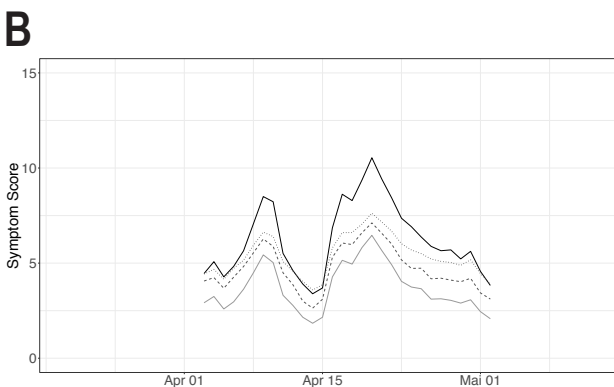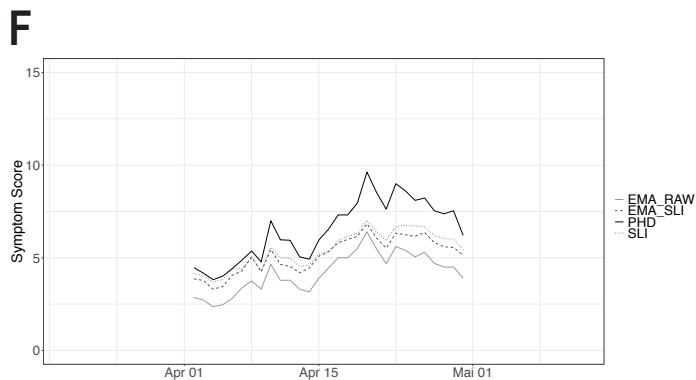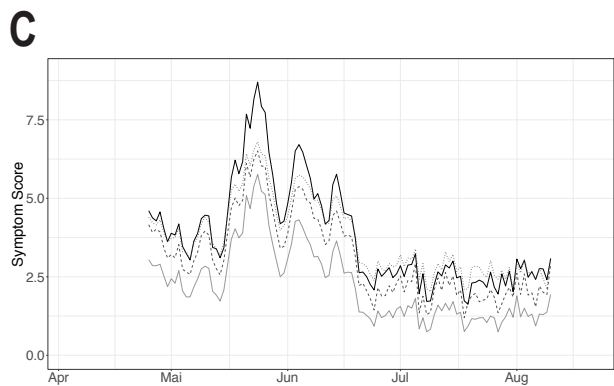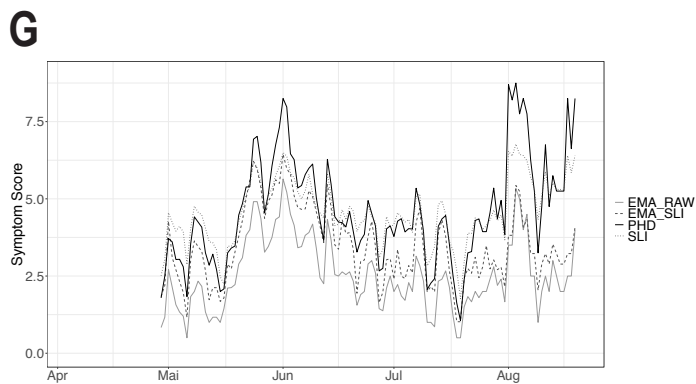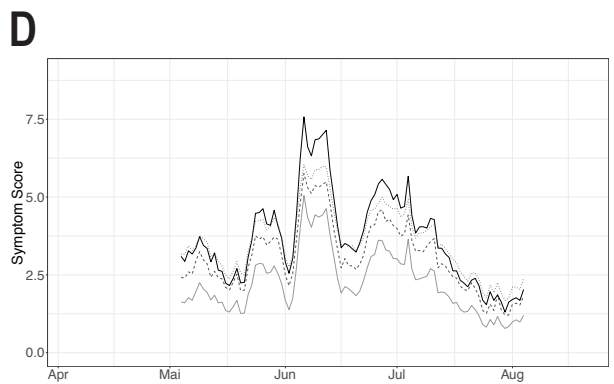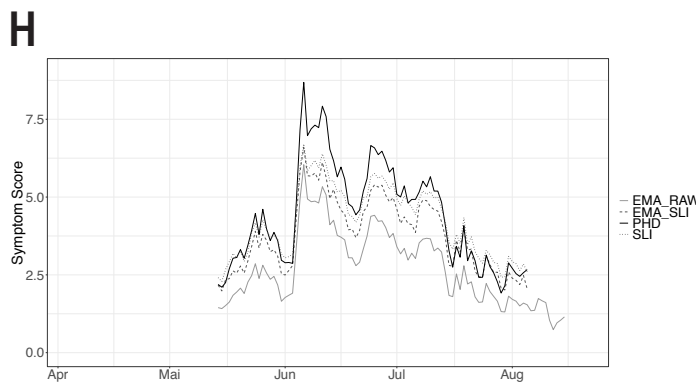

Supplement: Multimedia Appendix 8 [file jmir_v22i2e16767_app8.pdf]
